# Germination Season and Watering Regime, but Not Seed Morph, Affect Life History Traits in a Cold Desert Diaspore-Heteromorphic Annual

**DOI:** 10.1371/journal.pone.0102018

**Published:** 2014-07-11

**Authors:** Juan J. Lu, Dun Y. Tan, Jerry M. Baskin, Carol C. Baskin

**Affiliations:** 1 Xinjiang Key Laboratory of Grassland Resources and Ecology & Ministry of Education Key Laboratory for Western Arid Region Grassland Resources and Ecology, College of Grassland and Environment Sciences, Xinjiang Agricultural University, Urümqi, China; 2 Department of Biology, University of Kentucky, Lexington, Kentucky, United States of America; 3 Department of Plant and Soil Sciences, University of Kentucky, Lexington, Kentucky, United States of America; Cairo University, Egypt

## Abstract

Seed morph, abiotic conditions and time of germination can affect plant fitness, but few studies have tested their combined effects on plasticity of plant life history traits. Thus, we tested the hypothesis that seed morph, germination season and watering regime influence phenotypic expression of post-germination life history traits in the diaspore-heteromorphic cold desert winter annual/spring ephemeral *Diptychocarpus strictus*. The two seed morphs were sown in watered and non-watered plots in late summer, and plants derived from them were watered or not-watered throughout the study. Seed morph did not affect phenology, growth and morphology, survival, dry mass accumulation and allocation or silique and seed production. Seeds in watered plots germinated in autumn (AW) and spring (SW) but only in spring for non-watered plots (SNW). A high percentage of AW, SW and SNW plants survived and reproduced, but flowering date and flowering period of autumn- vs. spring-germinated plants differed. Dry mass also differed with germination season/watering regime (AW > SW > SNW). Number of siliques and seeds increased with plant size (AW > SW > SNW), whereas percent dry mass allocated to reproduction was higher in small plants: SNW > SW > AW. Thus, although seed morph did not affect the expression of life history traits, germination season and watering regime significantly affected phenology, plant size and accumulation and allocation of biomass to reproduction. Flexibility throughout the life cycle of *D. strictus* is an adaptation to the variation in timing and amount of rainfall in its cold desert habitat.

## Introduction

Plants that germinate at different seasons are subjected to different biotic and abiotic environmental conditions, which may have consequences on the life cycle. For example, in *Campanula* (*Campanulastrum*) *americana* (Campanulaceae) there are two germination seasons, with resulting consequences on the life cycle [1]. That is, plants from seeds that germinate in late summer or autumn overwinter as rosettes, are vernalized during winter and flower, set fruit and die the following summer; they behave as winter annuals. On the other hand, plants from seeds that germinate in spring grow during summer, are vernalized during winter and flower, set fruit and die the second summer; they behave as strict biennials. Thus, germination timing can influence the expression of post-germination life history characters [2]–[4].

Seed heteromorphism, i.e. the production on the same plant of seeds/fruits (sometimes with accessory parts) that differ in morphology and ecology [5], may play a role in determining when the seeds germinate [6], [7]. In many cases, the two or more seed morphs have different dormancy/germination behaviors and also give rise to plants that differ in life history characteristics [5]. Thus, seed heteromorphism may allow escape from the negative effects of sib competition [8] and reduce the risk of failure under temporal environmental uncertainty [9].

**Figure 1:**
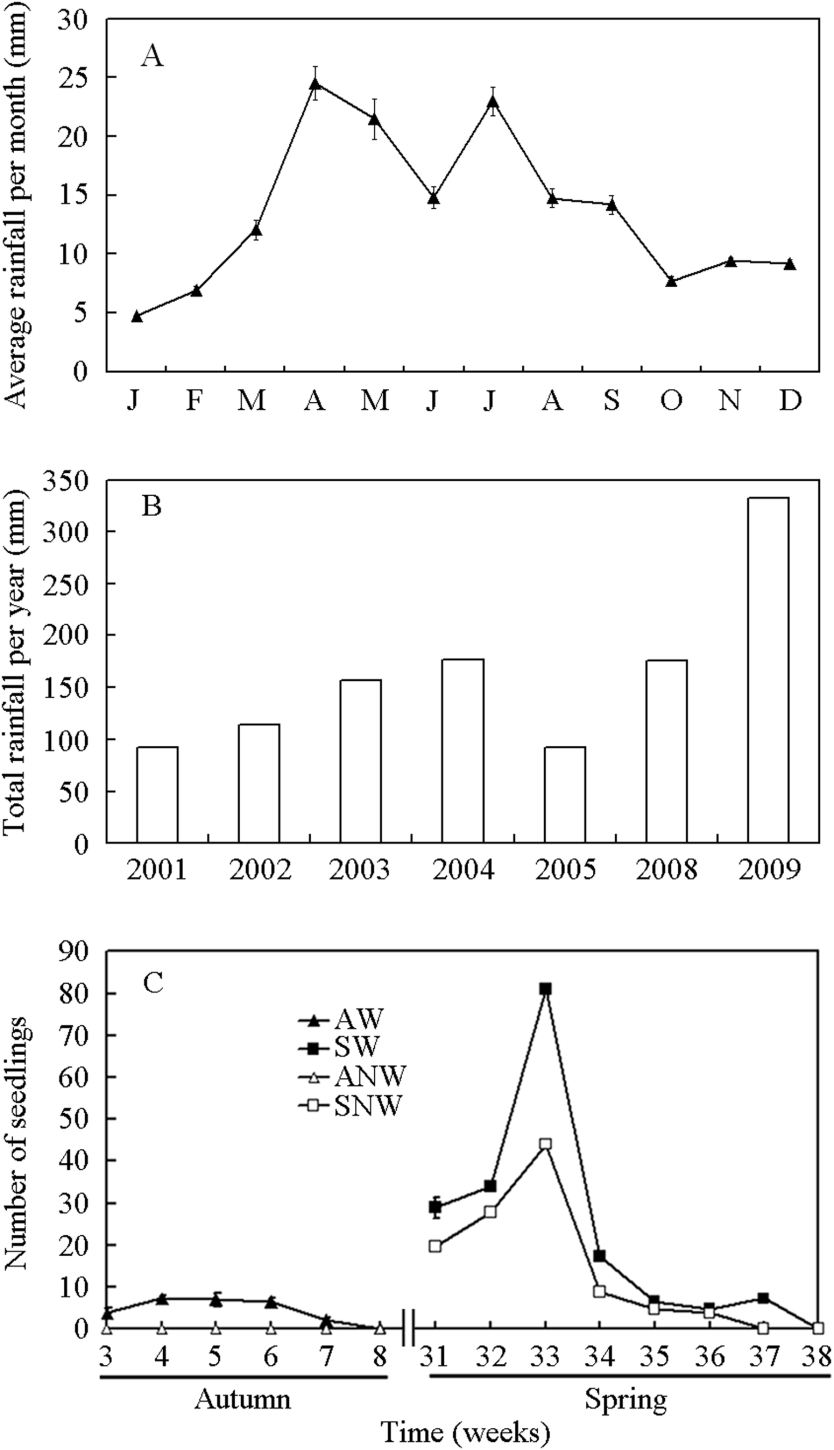
Comparison of monthly (A) and annual (B) rainfall in Urümqi, China, for 2001–2005, 2008, and 2009 (data from Xinjiang Agricultural Weather Station located near the experimental garden study plots); (C) Number of seedlings of *Diptychocarpus strictus* emerged (mean ±1 s.e.) each week in watered and non-watered plots in the experimental garden during autumn 2008 and spring 2009. AW, autumn-germinating plants watered; SW, spring-germinating plants watered; ANW, autumn-germinating plants not-watered; SNW, spring-germinating plants not-watered. In (A) and (C), only standard errors ≥0.39% and ≥2.31% are shown, respectively.

In hot deserts, such as the Sonoran and Negev, variation in timing and amount of precipitation can affect seed germination and growth of the resulting plants [10]–[12]. Similarly, in the cold Junggar Desert of northwest China, precipitation variability (Fig. 1A,B) can create the opportunity in some years for species to germinate in autumn as well as in spring. Additionally, year-to-year variability in precipitation may cause differences in germination timing within a season between years. Since earlier germination often has been linked to higher fitness in field studies [2], [3], [7], germinating early in years when it is possible to do so might be advantageous to the plant. However, germinating in a different season or later within a given season due to natural variation in rainfall or to climate change might require different life history, growth and resource allocation traits.

*Diptychocarpus strictus* (Fisch. ex Bieb.) Trautv. (Brassicaceae) occurs in Central Asia (including northwest China), Iran, Turkey and Caucasia [13]. In China, the species is found only in the cold desert region of northern Xinjiang, where it is a very common ephemeral (annual) species in autumn (in some years) and early spring (in most years) in the Junggar Desert. Individual plants produce either purple or white flowers, and all plants produce dimorphic fruits/seeds [14]. Fruits produced on the upper part of an infrutescence (upper siliques) are dehiscent at maturity, and the seeds within them are prominently winged and contain lots of mucilage. Those produced on the lower part of an infrutescence (lower siliques) are indehiscent, and their seeds are nearly wingless and essentially without mucilage. Thus, seeds from upper siliques and intact lower siliques are the germination and dispersal units of this species [14].

Since *D. strictus* produces dimorphic seeds and is likely to be under stressful conditions in its natural habitat, including a short period of time to complete its life cycle and limited water supply, we hypothesized that seed morph, germination season and watering regime would influence the phenotypic expression of its post-germination life history characters. More specifically, we hypothesized that (1) plants derived from different seed morphs that germinate in the same season or under the same watering regimes would differ in life history traits, and (2) germination season and watering regime would affect the expression of plasticity in the major life history traits. To test these two hypotheses, we compared phenology, growth and morphology, survival, biomass accumulation and allocation and silique and seed production of (1) autumn- vs. spring-germinating (AW vs. SW) plants, and (2) spring-germinating watered vs. spring-germinating not-watered (SW vs. SNW) plants derived from each of the two seed morphs of *D. strictus*.

## Materials and Methods

### Ethics approval

No specific permits were required for the described field studies. The location is not privately-owned or protected in any way, and the field studies did not involve endangered or protected species.

### Seed collection

Freshly-matured khaki-colored siliques that were dispersing naturally were collected from approximately 3000 individuals in June 2008, separated into upper and lower fruits and stored in paper bags at room conditions (18–30°C, 20–30% RH) until used.

The seed-collection site is located in the gravel desert in the vicinity of Urümqi on the southern edge of the Junggar Basin of Xinjiang Province in northwest China (43°48′N, 87°32′E, 850 m a.s.l.). This area of the Junggar Desert is dominated by species of Amaranthaceae (including Chenopodiaceae) and Asteraceae and has gravelly grey desert soil and a continental climate. Mean annual temperature is 6.8°C, and the extreme temperatures of the coldest (January) and hottest (July) months are −32.8°C and 40.5°C, respectively. Average annual rainfall can vary greatly between months and years (Fig. 1A, B), and the snow that falls in winter begins to melt in March or April. Annual potential evaporation is >2000 mm [15].

### Seedling growth rate assay

On 2 September 2008, 600 seeds (50 seeds × two seed morphs × six replicates) were germinated in Petri dishes filled with wet filter papers at optimum conditions (15/2°C, in light  = 12 h each day, ca. 100 umol m^−2^ s^−1^, 400–700 nm, cool white fluorescent light) [14]. Then, on 6 September 2008, 400 seedlings (ten seedlings per pot × two seed morphs ×20 pots) that germinated on the same day were transplanted to a greenhouse (20–25°C) into pots (18 cm deep and 20 cm in diameter) filled with soil from the natural habitat of *D. strictus*. The soil was watered daily throughout the experiment. After 0 (i.e. day of transplanting) 5, 10, 15 and 20 days of growth, 80 seedlings (two seedlings per pot × two seed morphs ×20 pots) were harvested and their roots washed free of soil. Fresh mass of their roots and shoots of the five age groups was determined using a Sartorius BS210S electronic-balance (0.0001 g). Then, the seedlings were oven-dried at 80°C for 48 h and their roots and shoots weighed using the Sartorius analytical balance. After 20 days of growth, the seedlings had reached the four-leaf stage, which was considered to be the end of the seedling growth stage and beginning of the rosette stage.

### Effect of seed morph and germination season/watering regime on flexibility of life history traits

**Figure 2:**
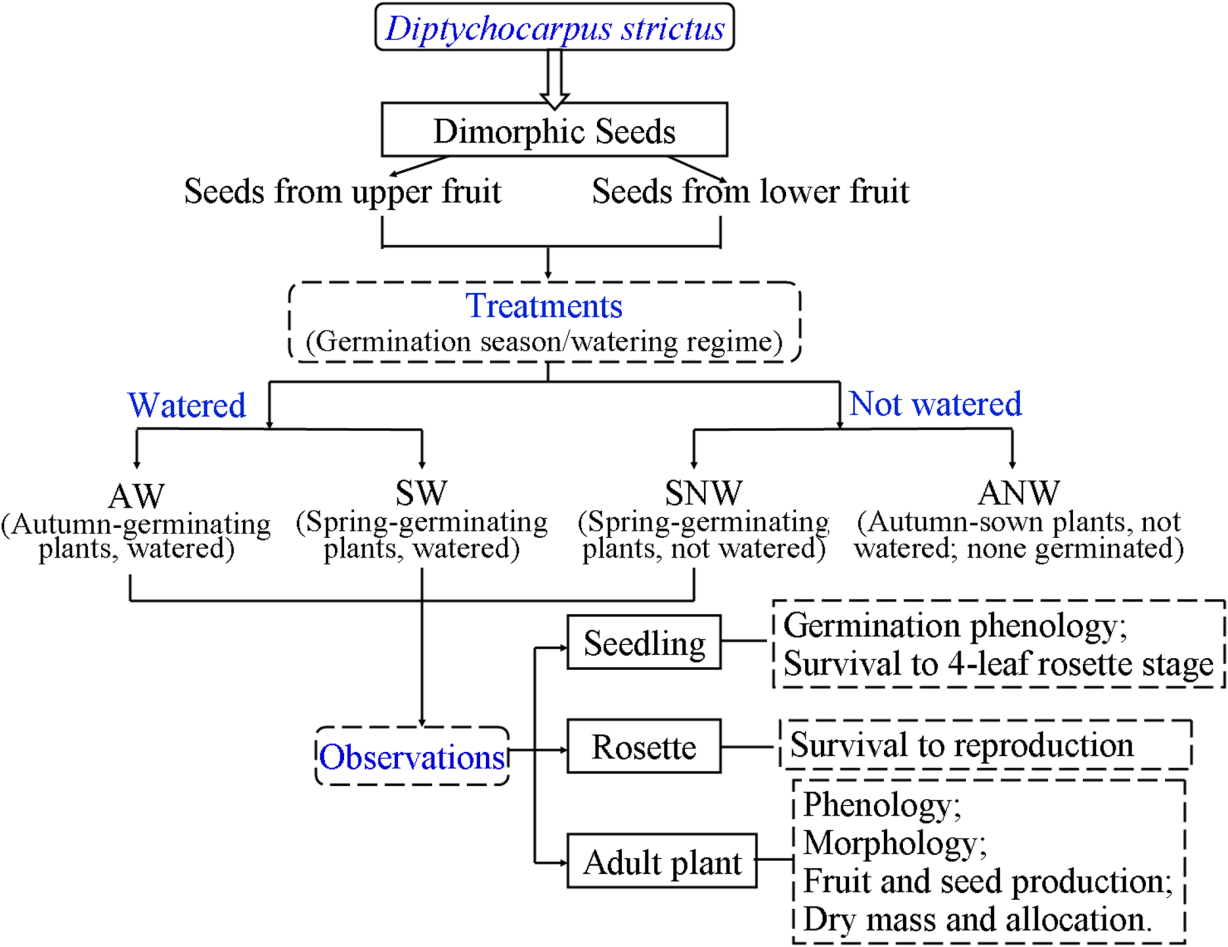
Experimental design of study on *Diptychocarpus strictus*.

On 23 August 2008, 9600 seeds (400 seeds × two seed morphs × two watering regimes × six replicates) were sown on bare soil in plots (1.5 m×0.8 m) in the experimental garden located on the campus of Xinjiang Agricultural University, Urümqi, China, in the southernmost part of the Junggar Desert. In the watered plots, the soil was watered to field capacity every 3 days to ensure that water was not a limiting factor for germination and growth of resulting plants. Plots were not watered during winter when the soil was frozen. In the non-watered plots, the soil received water only via natural rainfall and snowmelt. At 7-day intervals from August 2008 to June 2009, germinated seeds (seedlings) were counted and marked. The overall experimental design is shown in Fig. 2.

A total of 490 plants (51 AW plants from seeds of upper siliques +31 AW plants from seeds of lower siliques +102 SW plants from seeds of upper siliques +102 SW plants from seeds of lower siliques +102 SNW plants from seeds of upper siliques +102 SNW plants from seeds of lower siliques) was used to observe phenology. Also, a subset of 60 similarly-sized seedlings (six plots × five plants/plot × two seed morphs) each for AW, SW and SNW were marked to determine the other life history traits (i.e. survival, morphological characters, silique and seed production and dry mass accumulation and allocation).

#### Phenology

Emergence date (number of days since sowing until all seeds in a treatment had emerged), flowering date (number of days since sowing until all plants in a treatment had flowered), fruiting date (number of days since sowing until occurrence of the first green fruit on all plants in a treatment) and maturation date (number of days since sowing until the first fruit of all individuals in a treatment had turned a khaki-color and was ready to disperse naturally) were determined. Then, growth period (interval from emergence to final height), flowering period (interval from first flower that bloomed to last flower that withered in each treatment), fruiting period (interval from appearance of the first fruit to maturation of last fruit in each treatment) and post-germination life span (interval from emergence of the first individual to death of the last individual in each treatment) were obtained using the number of plants described above.

#### Survivorship

The number of seedlings surviving from germination to the four-leaf rosette stage and the number surviving from this stage to reproduction were monitored.

#### Morphological characters

Plant height (H, from soil surface to highest apical meristem), branch length (BL, the largest branch on main stem) and number of branches (BN, >1 cm in length on main stem) were measured. The average height growth rate per week (H) over the post-germination life span was calculated using the formula H = h/l (cm· week^−1^), where h is plant height at maturity and l life period. The diameter of each rosette also was recorded on the day the seedlings reached the four-leaf stage, which is the beginning of the rosette stage.

#### Silique and seed production

At harvest, number of infructescences per individual, fruits (siliques) per infructescence, fruits per individual, upper and lower siliques per infructescence and per individual, seeds per upper and lower silique and seeds of upper and lower siliques per individual were determined. Number of upper and lower siliques per infructescence (N) was estimated by dividing total number of upper and lower siliques per individual (N_t_) by number of infructescences per individual (N_i_), i.e. N = N_t_/N_i_. The total number of fruits per infructescence and per individual was estimated by summing the number of upper and lower siliques per infructescence and per individual. Number of seeds per upper silique and per lower silique was estimated by averaging the sum of the number of seeds in one silique each taken from the bottom, middle and top parts of each plant (i.e. three siliques per plant). Total number of seeds per plant was calculated by multiplying number of siliques by average number of seeds per silique.

#### Dry mass accumulation and allocation

The mature plants within each treatment were separated into root, stem, leaves and upper and lower fruits (including their pericarps and seeds). Then, all parts were oven-dried at 80°C for 48 h and weighed using a Sartorius BS210S electronic-balance (0.0001 g). Total biomass is vegetative (i.e. roots, stems and leaves) plus reproductive (i.e. upper and lower siliques) biomass. Allocation to roots, stems, leaves and reproductive organs was expressed as a percentage of the total dry mass.

### Statistical analysis

For data from the seedling growth assay, a repeated measurements ANOVA was used to test the effects of seed morph, growth days and their interaction on fresh mass, dry mass and root: shoot ratio of seedlings, with growth days as a within-subjects factor and seed morph as a between-subjects factor [16].

Pearson correlations were initially calculated for data from the garden experiment, including emergence date, flowering date, fruiting date, maturation date, growth period, flowering period, fruiting period, post-germination life span, survival percentage, percentage survival to reproduction, plant height at maturity, height growth rate per week, number of branches, branch length, number of upper and of lower siliques per individual, number of seeds of upper and of lower siliques per individual, total number of seeds per individual, number ratio and mass ratio of upper/lower siliques and of their seed ratio. There were significant correlations among most of the above variables (Table S1). Thus, only the important ones (i.e. emergence date, flowering date, fruiting date, flowering period, post-germination life span, survival percentage, percentage survival to reproduction, plant height, number of upper and lower siliques per individual, number of seeds of upper and lower siliques per individual, number ratio and mass ratio of upper/lower siliques and of their seed ratio) are shown in the results.

Then, the above important variables were analyzed as dependent variables with a two-way ANOVA. Seed morph and germination season/watering regime were considered fixed effects, and all interaction terms were included. Also, seedling size (i.e. diameter at four-leaf rosette stage) was used as a covariate to minimize the effects of variation in initial size among seedlings on dependent variables in these analyses.

If seed morph and interaction between seed morph and germination season/watering regime had no significant effects on dependent variables in the above two-way ANOVA analyses, data for seeds of upper and lower siliques were pooled. For the garden experiment, lack of seed germination in autumn in the non-watered treatment further complicated statistical analyses, and thus a one-way ANOVA was used to test for significant differences among the three germination season/watering regimes (AW, SW and SNW) in the above important variables.

Data were arcsine (percentage data) or log_10_ (other data) transformed as needed before analysis to approximate normal distribution and homogeneity of variance to fulfill assumptions of a one-way ANOVA. If variance of transformed data was still not homogenous, treatment differences in these characteristics were assessed using the Kruskal-Wallis non-parametric test. Tukey's HSD test was performed for multiple comparisons to determine significant differences among treatments. Correlative analyses also were used to determine the relationship between dry mass of reproductive organs and plant height and between dry mass allocation to reproductive organs and plant height. Statistical tests were conducted at *P* = 0.05. All data analyses were performed with the software SPSS 13.0 (SPSS Inc, Chicago, Illinois, USA).

## Results

### Seedling growth rate assay

Fresh mass (*F* = 563.59, *df* = 4, *P*<0.001), dry mass (*F* = 2343.00, *df* = 4, *P*<0.001) and root: shoot ratio (*F* = 488.18, *df* = 4, *P*<0.001) increased with growth time after germination (data not shown). However, seed morph did not have an effect on any of these variables.

### Effect of seed morph and germination season/watering regime on flexibility of life history traits

Germination season/watering regime had significant effects on the life history traits of offspring (Table 1). However, none of the traits was significantly affected by seed morph (*P*>0.05) or any interaction (*P*>0.05). Thus, data from seeds of upper and lower siliques were pooled and analyzed with one-way ANOVA.

**Table 1:** Summary of a two-way ANOVA showing the effects of different germination season/watering regime treatments (T) (i.e. AW, SW and SNW) and seed morph (S) on phenology, survival, morphology and number and mass of siliques and of seeds from *Diptychocarpus strictus* offspring.

	I	T	S	T×S
*df*	1	2	1	2
Phenology				
Emergence date	6.31*	8964.58**	3.36	0.13
Flowering date	0.49	120.98**	1.78	1.89
Fruiting date	2.39	37.17**	0.59	1.04
Flowering period	9.77**	63.06**	0.92	1.71
Post-germination life span	0.97	5258.65**	3.84	0.10
Survival percentage, S′/N(%)	3.68	10.63**	1.58	2.52
Percentage survival to reproduction, R/S′	0.58	1.70	2.12	2.38
Morphology				
Plant height at maturity	3.63	249.98**	0.12	1.51
Number per individual plant				
Upper siliques	2.31	70.45**	0.17	0.61
Lower siliques	0.39	56.59**	0.04	0.37
Seeds from upper siliques	3.77	50.79**	0.02	0.23
Seeds from lower siliques	1.56	26.47**	0.00	0.10
Ratio				
Upper/lower silique number ratio	0.33	13.18**	0.03	0.26
Seed number ratio from upper/lower siliques	0.51	18.29**	0.04	0.07
Upper/lower silique mass ratio	2.81	32.53**	0.23	0.25
Seed mass ratio from upper/lower siliques	0.64	18.97**	0.15	0.14

Initial size (I) refers to the diameter of seedling at four-leaf rosette stage, which was a covariate (see text). The *F* ratios given are based on Type III sums of squares. *P<0.05; **P<0.001. S′, number of plants that survived to four-leaf rosette stage during treatment; N, total number of germinated seedlings; R, number of surviving four-leaf rosette plants that reproduced during treatment.

#### Phenology

Germination season/watering regime affected key stages of the life cycle (Table 2). Seeds in the watered treatment germinated in autumn (AW) and in spring (SW), whereas in the non-watered treatment seeds germinated in spring (SNW) but not in autumn (ANW) (Fig. 1C). AW plants behaved as winter annuals and SW and SNW plants as spring ephemerals.

**Table 2:** Effect of germination season/watering regime on phenology, survival, morphology and silique and seed production of plants in *Diptychocarpus strictus* (mean ±1 s.e.).

	AW	SW	SNW
Phenology			
Emergence date (d)	34.90±0.25^a^	211.18±0.48^b^	214.00±0.60^c^
Flowering date (d)	248.65±0.57^a^	256.78±0.39^b^	263.87±0.25^c^
Fruiting date (d)	277.52±0.33^a^	280.35±0.41^b^	283.62±0.32^c^
Flowering period (d)	40.75±0.32^b^	34.30±0.22^a^	32.75±0.39^a^
Post-germination life span (l, d)	242.63±0.87^b^	72.37±0.37^a^	71.33±0.32^a^
Survival percentage, S′/N (%)	100.00±0.00^b^	98.78±0.46^b^	94.77±1.26^a^
Percentage survival to reproduction, R/S′ (%)	100.00±0.00^a^	100.00±0.00^a^	98.61±0.94^a^
Morphology			
Plant height at maturity (H, cm)	52.99±0.40^c^	34.61±0.51^b^	24.24±0.62^a^
Silique and seed production per individual			
Number of upper siliques	89.73±3.39^c^	23.67±1.21^b^	7.47±0.55^a^
Number of lower siliques	48.88±1.89^c^	15.58±0.83^b^	7.83±0.48^a^
Number of seeds from upper siliques	2890.12±127.01^c^	692.02±37.26^b^	200.77±15.00^a^
Number of seeds from lower siliques	964.90±51.63^c^	283.23±16.91^b^	156.80 ±10.30^a^

AW, autumn-germinating plants watered; SW, spring-germinating plants watered; SNW, spring plants not-watered. S′, number of plants that survived to four-leaf rosette stage during treatment; N, total number of germinated seedlings; R, number of surviving four-leaf rosette plants that reproduced during treatment; H, plant height at maturity; l, life period. Different lowercase letters (a, b, c) indicate significant differences (Tukey's HSD, *P* = 0.05).

*Note*: Dates for emergence, flowering and fruiting are number of days (d) since sowing. Flowering interval, interval from blooming of first flower to withering of last flower; post-germination life span, interval from emergence to death.

The interval from seedling emergence to flowering in AW (214 days) plants was significantly longer than that in SW (46 days) and in SNW (50 days), which were not significantly different (Table 2). Also, flowering period was significantly positively correlated with plant size (plant height) at maturity (Table S1). Although AW plants had a longer post-germination life span than SW and SNW, the proportion of the reproductive period for the whole post-germination life span of AW was significantly shorter than the proportions for SW and SNW, which were not significantly different (Table 2).

#### Survival

A high percentage of seedlings from all treatments survived and reproduced (Table 2). All plants from the 82 seeds that germinated in autumn 2008 reproduced in spring 2009, and 98–100% of plants from the 408 seeds that germinated in early spring 2009 reproduced in late spring 2009.

#### Morphological characters

Plant size (plant height) was highly significantly different between autumn- and spring-germinating plants (Table 2). Thus, order of plant height was AW > SW > SNW.

#### Silique and seed production

Germination season/watering regime significantly affected all parameters of siliques and seeds, except seed number per lower silique (Table 2). SW and SNW had significantly fewer upper and lower siliques per individual than AW (Table 2). Also, plant size (plant height) was significantly positively correlated with number of upper siliques and their seeds (Table S1). Thus, variation in plant size strongly influenced reproductive output.

#### Dry mass accumulation and allocation

**Figure 3:**
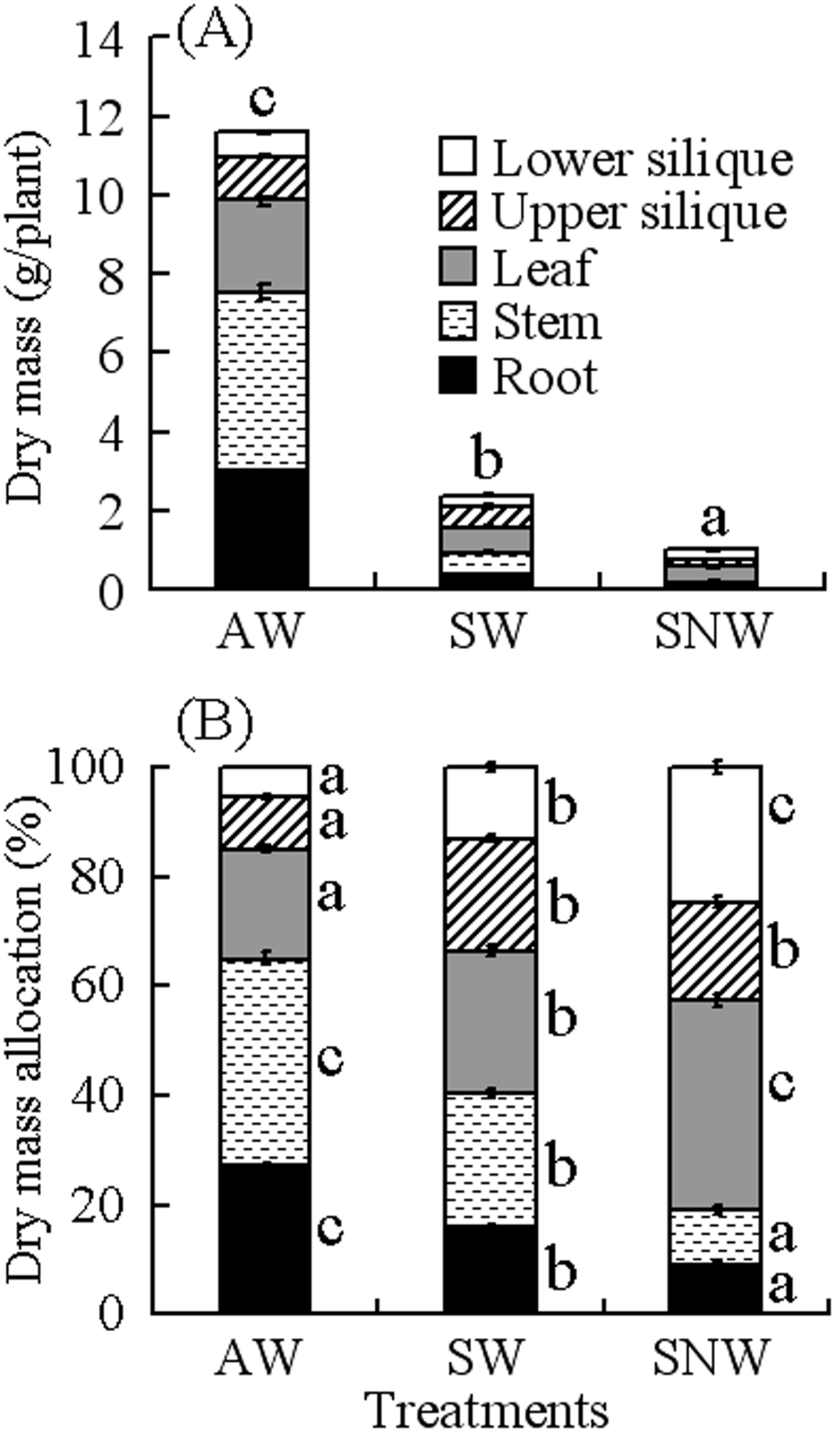
Effect of germination season/watering regime on dry mass accumulation (A) and allocation (B) (mean ±1 s.e) in *Diptychocarpus strictus*. AW, autumn-germinating plants watered; SW, spring-germinating plants watered; SNW, spring-germinating plants not-watered. Bars with different letters for total dry mass (A) or for portions of bars for dry mass allocation (B) indicate significant difference in multiple range comparison (Tukey's HSD, *P* = 0.05).

Total dry mass of plants was about 11.6, 2.4 and 1.0 g plant^−1^ in AW, SW and SNW, respectively (Fig. 3A), and dry mass of reproductive organs was 1.7, 0.8 and 0.4 g plant^−1^, respectively. Dry mass of reproductive organs also was significantly positively correlated with plant height (*r* = 0.88, *P*<0.001).

Allocation to roots and stems was higher in AW than in SW and SNW, with the opposite result for dry mass allocation to leaf and reproductive organs (Fig. 3B). Allocation of dry mass to reproduction was significantly negatively correlated with plant height (*r* = −0.81, *P*<0.001). The high proportion of dry mass allocated to reproduction resulted in a relatively small amount allocated to roots and stems.

**Figure 4:**
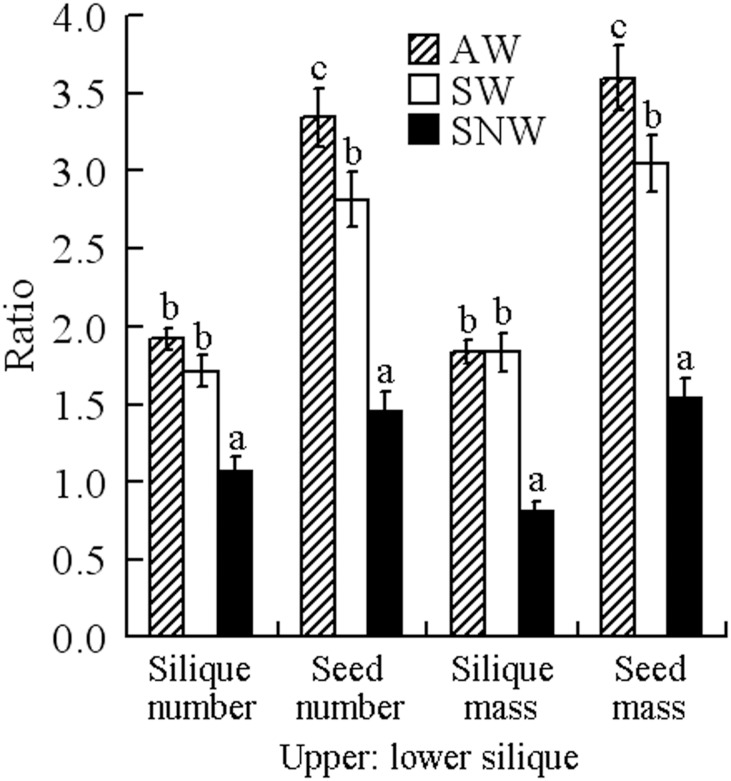
Effects of germination season/watering regime on number ratios and mass ratios of siliques and of seeds per individual (mean ±1 s.e.) in *Diptychocarpus strictus*. AW, autumn-germinating plants watered; SW, spring-germinating plants watered; SNW, spring-germinating plants not-watered. Bars with different letters within each of the four ratio categories are significantly different in multiple range comparison (Tukey's HSD, *P* = 0.05).

In addition, number ratio and mass ratio of upper/lower silique was significantly affected by watering regime but not by germination season, whereas number ratio and mass ratio of their seeds was significantly affected by both germination season and watering regime (Fig. 4).

## Discussion

Our first hypothesis that plants derived from the two seed morphs that germinate in the same season or under the same watering schedule would differ in life history traits was not supported. However, our results indicate that post-germination life history traits of *D. strictus* differed greatly between AW and SW (germination season) and between SW and SNW (watering regime). Thus, our second hypothesis that germination season and watering regime would significantly affect the expression of plasticity in all major post-germination life history traits was confirmed. Although many studies have tested the effects of seed morph [5], [17], [18] or germination season [4], [19]–[21] on a limited number of plant life history traits such as survival, growth and reproduction, ours is the first detailed account of its kind on a cold desert annual/ephemeral.

One reason why plants of *D. strictus* derived from the two seed morphs of unequal size did not differ in life history traits is that seed masses are the same after the wings and mucilage are removed from them [14]. Further, since the endosperm in Brassicaceae is very thin, size of the embryo also would not differ between the two seed morphs. Further, growth rates of the plants derived from the two seed morphs obviously were the same throughout their life cycle since they did not differ significantly in final size (Table 1).

The germination behavior of *D. strictus* seeds allows them to germinate in both autumn and spring. Thus, the plants then must also have the ability to (1) survive through the winter in a vegetative state under the snow and through dry autumns and springs (Fig. 1A), and (2) time flowering and allocate resources appropriately despite the different set of conditions. Survival in autumn- and spring-germinating plants of *D. strictus* was higher than pre-reproductive survival of autumn and spring germinants of the weedy winter annual/ephemeral *Diplotaxis erucoides* [4], in which survival varied with disturbance regimes and between and within cohorts [22]. In *Arabidopsis thaliana*, 96% of the rosettes (not-watered) of both early- and late-autumn germinants survived the winter and bolted (initiation of reproduction), whereas no spring-germinating plants survived to this stage except those that received supplemental watering [23].

In *Arabidopsis thaliana*, time to flowering is accelerated by chilling the seeds or by chilling the rosettes [24], [25], and chilling can break dormancy in dormant seeds and induce it in nondormant seeds [26], [27]. Further, prolonged chilling of seeds of *A. thaliana*, independently of their dormancy status (i.e. dorment vs. nondormant), shortened the time to bolting (reproduction) [28]. Thus, the environment experienced by the seed stage of this annual plant species affects plant life history beyond (and independently of) germination date [28]. In our study, the post-germination life span of SW and SNW plants of *D. strictus* was much shorter than that of AW plants (Table 2). We speculate that the short life spans of the SW and SNW plants could be due at least in part to chilling (vernalization) of the nondormant seeds during the long period between sowing in summer and germination in spring [14]. Thus, time to reproduction would be accelerated and consequently the life cycle shortened, which would be adaptive in the Junggar Desert where rainfall is unpredictable in spring (Fig. 1A).

Autumn germination in *D. strictus* greatly increased the mean fitness of individuals in the population, given that rosette survival percentage was high during the winter. The numerical increase in fitness between autumn and spring germinators is consistent with that reported for *Diplotaxis erucoides*, for which the number of seeds produced by autumn-germinating plants was 3 to 10 times higher than that produced by spring germinators [4], [22]. Likewise, autumn-germinating plants of *Lactuca serriola* produced about 10 times more seeds per individual than spring- and summer-germinating plants [20].

The larger plants of *D. strictus* allocated the least proportion of biomass to reproduction and the smallest plants the greatest proportion. However, the absolute amount of biomass (g) increased with plant size. Likewise, total biomass allocated to reproduction in the three South African desert ephemerals *Dimorphotheca sinuata*, *Ursinia calenduliflora* and *Heliophila pendula* increased with plant size, whereas proportion of total biomass allocation to reproduction increased with decrease in plant size, which was related to time of germination and thus to length of growing season [29]. Thus, like *D. strictus* plants those of these three species that germinated in autumn were larger, produced more total reproductive biomass and allocated a smaller proportion of total biomass to reproduction than spring-germinating plants.

We suggest that the increase in proportion of biomass allocated to reproduction in *D. strictus* in a short growing season (AW vs. SW) and under limiting soil moisture (SW vs. SNW) can be interpreted as stress responses. This interpretation is supported by the results of Lu et al. that stressful growth conditions caused a shift of proportionally more biomass to reproduction and increased the proportion of the “low risk” (i.e low dispersal – high dormancy) lower indehiscent diaspores in *D. strictus* [30]. We interpret these plastic responses in life history traits to stress to be adaptive in that they increase fitness (i.e. number of seeds produced per gram of biomass). For *D. strictus*, the negative effects of delaying germination until spring (short growing season) and of an inadequate water supply for maximum growth of spring germinators probably are reduced by its ability to allocate a higher proportion of resources to reproduction under these stressful conditions.

The importance of seed/fruit heteromorphism in *D. strictus* is not due to differences in germination ecophysiology of the dimorphic seeds [14], response of plants to stressful conditions [30] or life history (present study) but to release of the winged, mucilaginous seeds from upper dehiscent siliques at maturity and retention of the essentially nonwinged, nonmucilaginous seeds in a state of enforced dormancy for >1 year within the indehiscent lower siliques [14]. These seed/fruit characteristics mean that the seeds from the upper siliques have a high-risk strategy (HR) and those from the lower siliques a low-risk strategy (LR) for both dispersal ability and degree of dormancy (HR-LR). Thus, the winged seeds in the upper dehiscent fruits have good dispersal ability and a short dormancy period (HR) and the ones in the lower indehiscent siliques poor dispersal ability and a long dormancy period (LR). This bet-hedging strategy [9] distributes plants in space (upper seed) and time (lower seed) and thus is an adaptation to the unpredictability of the cold desert environment.

A characteristic feature of the cold desert flora in the Junggar Desert is the presence of numerous species of ephemerals, many of which germinate in spring and complete their life cycle in 2 to 3 months [31]. In this cold desert, rainfall is highly variable among seasons and years, but generally rainfall is higher in spring than in autumn (Fig. 1A,B; [31]). Further, water from snowmelt increases water availability in spring. Thus, seeds of the winter annuals/ephemerals are more likely to germinate in spring than in autumn. However, sufficient precipitation in autumn occasionally occurs to stimulate seeds of this ecological group of species to germinate. Seeds of *D. strictus* is one member of the cold desert flora whose germination behavior allows them to germinate under natural rainfall in both seasons.

In conclusion, our study shows that there is great flexibility in the post-germination stages of the life cycle of *D. strictus*, which is an adaptation to the variation in timing and amount of rainfall in the unpredictable habitat of this cold desert winter annual/spring ephemeral. In particular, we have shown that fitness is affected by the season (autumn *vs*. spring) in which the seeds germinate and by the amount of rainfall in spring.

## Supporting Information

Table S1 **Pearson correlations among characters for AW, SW and SNW plants (from upper and lower seeds of the two flower-color morphs).** N  =  180. *P<0.05; **P<0.001.
(XLS)
